# Systematic review and meta-analysis of no show or non-attendance rates among telehealth and in-person models of care

**DOI:** 10.1186/s12913-025-12826-2

**Published:** 2025-05-09

**Authors:** Edwin Phillip Greenup, Daniel Best

**Affiliations:** Department of Health, Clinical Excellence Queensland, Queensland Health, Level 2, 15 Butterfield Street, Herston, Brisbane, QLD 4006 Australia

**Keywords:** No show, Fail to attend, Non-attendance, Attendance, Virtual care, Telehealth, Telemedicine

## Abstract

**Objectives:**

Comparisons of no-show rates between virtual and in-person models of care are commonly reported during evaluation, indicating how coveted improvements in attendance are to health service providers. This study seeks to synthesise the data from studies that make these comparisons, providing a more accurate indication of what may expected from the use of virtual care models for clinicians and administrators.

**Study design:**

Systematic review and meta-analysis of 45 retrospective cohort studies.

**Methods:**

A literature review involving five databases was conducted, identifying 441 articles for screening. 45 were included for further analysis. A Random effects model was used to calculate the effect size, with further analysis conducted to determine heterogeneity and publication bias.

**Results:**

The Random effects model estimated a reduced likelihood non-attendance in patients receiving virtual care compared to in-person groups *(OR = 0.61)*. An I^2^ indicated a high degree of heterogeneity among the studies analysed. The Fail-Safe N suggested that the results are robust and not significantly influenced by publication bias.

**Conclusions:**

The meta-analysis indicated that on average, telehealth models of care implemented since COVID-19 provide a moderate reduction in risk of patient non-attendance when compared to in-person alternatives.

**Supplementary Information:**

The online version contains supplementary material available at 10.1186/s12913-025-12826-2.

## Introduction

The COVID-19 pandemic led to a rapid increase in telehealth and the models of care implemented in response to the pandemic are now being evaluated [1–4]. These evaluations frequently describe the acceptance of telehealth by patients and clinicians in settings such as delivering stay-at-home outpatient care, making better use of hospital resources during the pandemic surge and supporting post-pandemic recovery [2].

A common metric in these evaluations is the comparison of attendance rates (also referred to as a non-attendance, failure to attend or no-show rate) between the new virtual model of care and a previously or concurrently offered in-person service [5]. This may be because well-designed virtual models are promoted as offering greater accessibility to patients. With the burden of travel removed, an improved attendance rate is considered a proxy measure of suitability and convenience of the appointment for the patient [2]. Several theoretical frameworks may support this belief including Fundamental Causation Theory in which patients from lower socioeconomic backgrounds may face more barriers, such as transportation or competing priorities, and Candidacy Theory, where patients may not identify the need for an appointment or feel it has relevance to them [6].

A new virtual service that demonstrates reduced rates of non-attendance can claim to be delivering benefit to the patient and service provider alike. This analysis seeks to synthesise these rates across the available literature. In doing so, it will determine if offering virtual care models influences a patient’s likelihood to attend their appointment. This may be useful to clinicians and administrators to better understand what they can expect to observe from well-implemented virtual care models of their own.

## Method

### Literature search

**Fig. 1:**
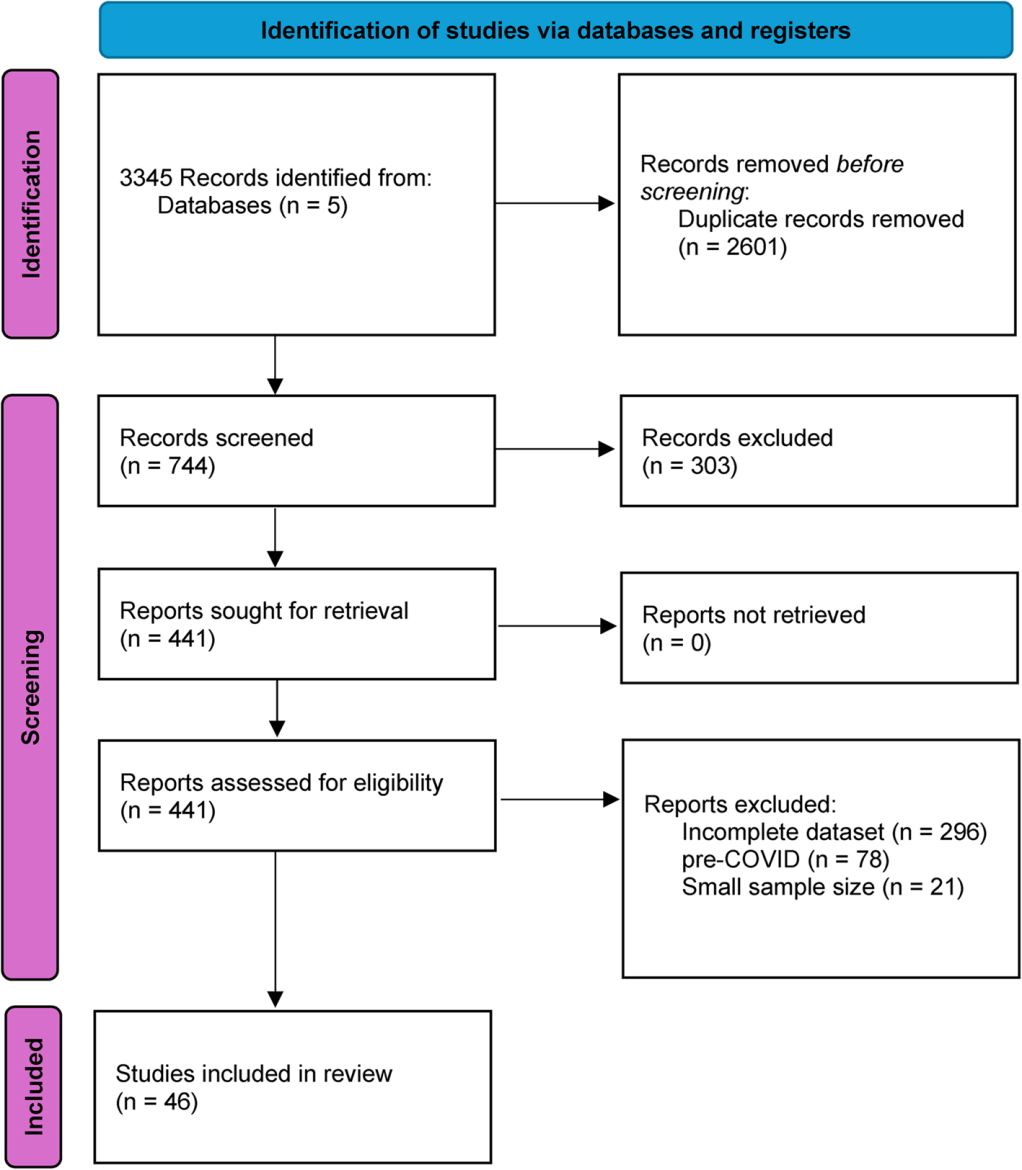
Identification of studies via databases and registers flow diagram [7]

A literature search was registered in PROSPERO in July 2023 *(reference: 447316)* and was conducted using terms non* attend*, fail* attend*,* did not attend*,* failed to attend*, no show*, non-appearance*, missed appointment* or patient attend* in articles published from January 2020 to June 2023 (see Supplementary material). The search was conducted in Ovid Medline, ProQuest Health Research, Joanna Briggs Institute, Cochrane Library and Embase. Both researchers followed a systematic process to rate articles for inclusion. Initially, article titles and abstracts were independently screened, with full texts of selected articles reviewed to further assess their eligibility. Both researchers rated articles based on pre-determined criteria to ensure consistency and minimise bias. Of interest were articles written in English describing an existing or newly implemented telehealth model of care which included a measure of attendance in the evaluation of both the telehealth and equivalent in-person modalities. Studies conducted prior to the COVID-19 pandemic were removed as were any studies with small frequencies of non-attendance of any modality type. The process followed to identify, screen, and include studies is further described in Fig. 1. Studies reporting fewer than five telehealth or in-person appointments (either attended or not attended) were removed from the study due to the difficulties in detecting the effect of appointment type in small samples. The relatively simple and unambiguous inclusion criteria produced no disagreement among researchers and consultation with a third reviewer was not required.

### Analysis and calculation of effect size

A random-effects model was used in the analysis. The analysis focused on the log odds ratio as the outcome measure. Heterogeneity statistics including I² and the Q statistic were calculated as were Model Fit Statistics and assessment for Publication Bias. Finally, the Rank Correlation Test for Funnel Plot Asymmetry, specifically using Kendall’s Tau was performed to further assess whether there is bias in the published studies.

## Results

**Fig. 2:**
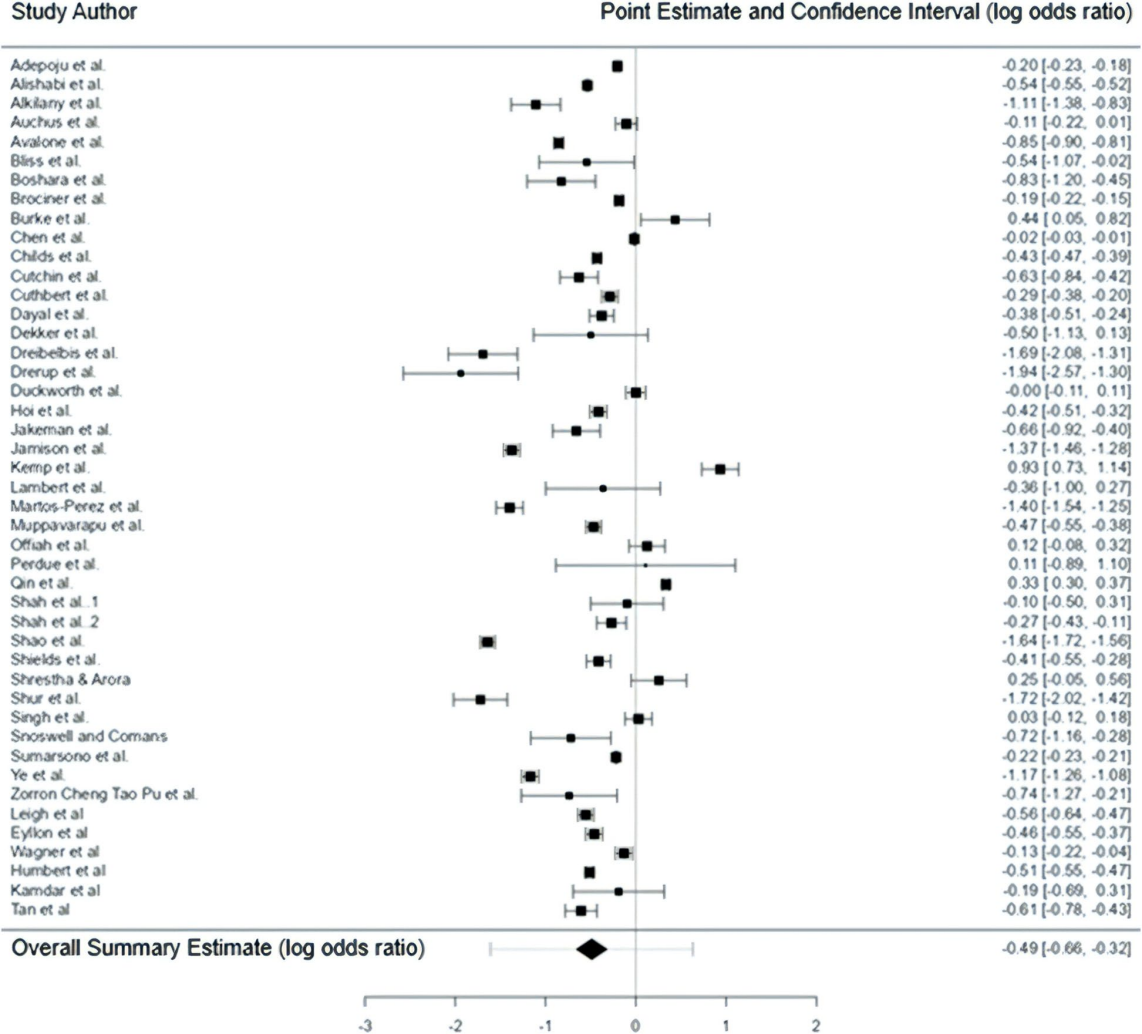
Forest plot graphical representation of the results of the meta-analysis, displaying the point estimates and confidence intervals from individual studies and overall summary estimate (log odds ratio)

A total of 45 studies were included in the model [8–52] (one study had a value larger than ± 3.27 which may be a potential outlier due to significant methodological differences in the context of this model and was removed from final analysis) [53]. The observed log odds ratios ranged from − 1.94 to 0.93, with most estimates being negative (84%). The estimated average log odds ratio based on the random-effects model was \hat{\mu} = -0.49 (95% CI: -0.66 to -0.32) or odds ratio of 0.61. See Table 1 and Fig. 2 and Supplement. Therefore, the average outcome differed significantly from zero (z = -5.62, *p* < 0.0001). This observed reduction in non-attendance in telehealth patients, with a p-value of < 0.0001, is not only statistically significant but also likely to be clinically significant, given the effect aligns with improvements in patient attendance and adherence to treatment frequently reported in primary studies. Evidence of economic benefits of telehealth models as a result of reduced non-attendance and increased cost efficiency were not made in any of the primary studies and could not be analysed further.

**Table 1:** Random-effects model (k = 45)

	**Estimate**	**se**	**Z**	**p**	**CI Lower Bound**	**CI Upper Bound**
Intercept	-0.489	0.0870	-5.62	< 0.001	-0.659	-0.319
	.	.	.	.	.	.

Tau² Estimator: Maximum-Likelihood

According to the Q-test, the true outcomes seem heterogeneous (Q(44) = 9622.12, *p* < 0.0001, tau² = 0.32, I² = 99.89%). A 95% prediction interval for the true outcomes is given by -1.61 to 0.63 (see Table 2). An examination of the studentised residuals revealed that none of the studies had a value larger than ± 3.26 and hence there was no indication of outliers in the context of this model. According to the Cook’s distances, none of the studies could be overly influential. Neither the rank correlation nor the regression test indicated any funnel plot asymmetry (*p* = 0.13 and *p* = 0.49, respectively).

**Table 2:** Heterogeneity statistics

Tau	Tau²	I²	H²	R²	Df	Q	*p*
0.565	0.3188 (SE = 0.0714)	99.89%	922.508	.	44.000	9622.117	< 0.001

The log-likelihood, deviance, AIC, BIC and AICc model fit statistics (see Table 3) support the adequacy of the model fit and the researchers believe that the model fit is considered acceptable.

**Table 3:** Model fit statistics and information criteria

	log-likelihood	Deviance	AIC	BIC	AICc
Maximum-Likelihood	-39.926	236.312	83.852	87.465	84.137
Restricted Maximum-Likelihood	-39.546	79.091	83.091	86.660	83.384

Fail-safe N analysis was performed to assess the risk of publication bias in this meta-analysis (see Table 4). The Fail-safe N value for this research was calculated at 80572.00. The researchers believe robust results such as these are not significantly influenced by publication bias.

**Table 4:** Publication bias assessment

Fail-safe *N*	*p*
80572.000	< 0.001

Fail-safe N Calculation Using the Rosenthal Approach

A Newcastle-Ottawa Scale (NOS) measurement of each primary study was performed to determine quality of selection, comparability and outcome results (M = 5.42, Med = 6) The observed values ranged from a minimum of 3 to a maximum of 8 (Range = 5).

**Fig. 3:**
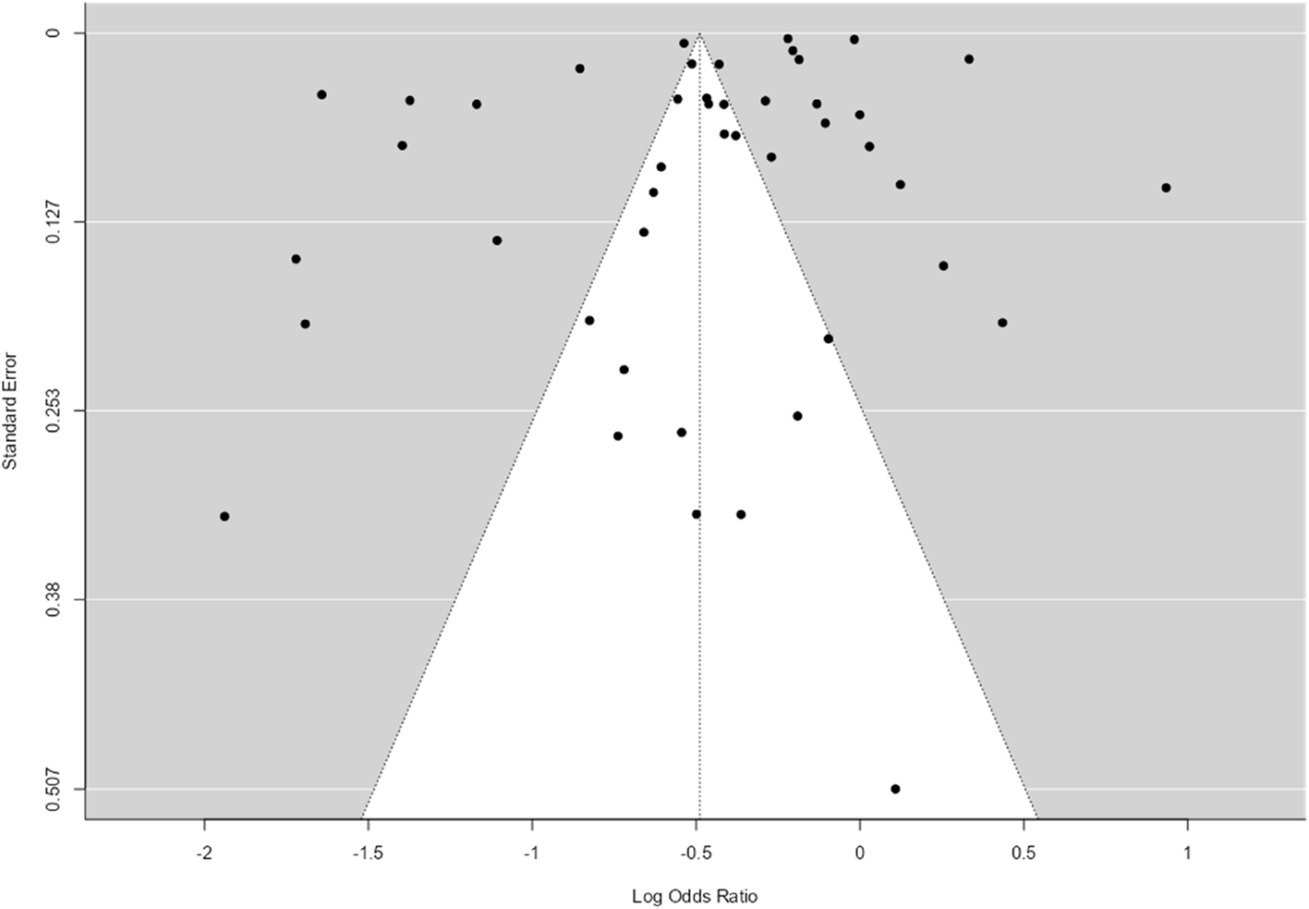
Rank correlation test for funnel plot asymmetry

Finally, neither the rank correlation nor the regression test indicated any funnel plot asymmetry (*p* = 0.13 and *p* = 0.49, respectively). See Table 5 and Fig. 3.

**Table 5:** Regression test for funnel plot asymmetry

Rank Correlation Test for Funnel Plot Asymmetry and Regression Test for Funnel Plot Asymmetry			
**Kendall’s Tau**	**p**	Z	P
0.158	0.130	-0.696	0.486

## Discussion

In many evaluations conducted since COVID-19, non-attendance rates in telehealth models of care are significantly reduced in contrast to the average rate of in-person comparators [8–15, 17–24, 27–30, 33–35, 38–44, 46–52]. This was reflected in the aggregated results of this meta-analysis. Although non-attendance can be caused by conditions present in both these modalities including patient-side (forgetfulness, competition for patient’s time and resources) and provider-side factors (poor communication, poor engagement with the patient, lack of flexibility), this reduction demonstrates that a well-designed telehealth model of care can remove some barriers to accessing treatment and provide a moderate effect in reducing non-attendance [54]. These barriers likely centre around the time and costs associated with physically attending an appointment.

Where non-attendance of telehealth appointments remains an issue, systematic factors were proposed such as socioeconomic status and the digital literacy of a region or group [13, 32, 38, 47, 51]. This contrasts with previous telehealth research which focussed on individual concerns about privacy or efficacy and technical difficulties [55]. This may be an indication that patients have become more familiar and accepting of telehealth appointments since the onset of the pandemic and large-scale studies reporting no reduction in telehealth non-attendance compared to in-person appointments are becoming less common [5].

### Limitations

Care was made during this study to compare only patient groups that attended with patient groups that did not for each modality. In some studies, patients who cancelled on the day of their appointment or were late may have been counted as not attending which complicated this comparison. Similarly, while most of these studies describe live, two-way video as the technology facilitating these models of care, some evaluations may use the telephone and include this under the definition of telehealth.

In evaluations where reductions in non-attendance were not observed, it is possible that these statistics were omitted or considered less relevant than other outcomes such as patient satisfaction, differences in how patients were managed or their outcomes.

Most of the studies included in this analysis were retrospective cohort studies. The measurement of non-attendance was not often the primary purpose of the study (i.e. the articles were typically focussed on whether or not telehealth was accepted proportionately, not whether or not it was offered proportionately). Although many primary studies used in this systematic review described reductions in non-attendance when the entire clinic converted from in-person to telehealth appointments, suggesting the appointment type produced this effect [14, 20, 22, 36, 37, 39, 42], the majority of studies describe clinics in which both appointment types were offered concurrently making the relationship between attendance and appointment type less clear.

Valuable information needed to determine the suitability of an article describing the effect of the appointment type was often not supplied (see Supplement). This would ideally include (i) how patients were selected for telehealth or in-person appointments and (ii) whether the telehealth workflow different in any other way to how in-person appointments are provided, such as additional reminders or training from the clinic. 

It is possible that patients who accepted the offer of a telehealth appointment were on average more comfortable with technology, more health literate, and therefore more likely to attend their appointment regardless of the appointment type. It is also possible that some newly established telehealth services offered patients different, more personal communication which enhanced the patient experience compared to in-person appointment recipients. This may have contributed to the observed non-attendance rate reduction.

Many of the studies took place when patients were experiencing lockdown conditions during the COVID-19 pandemic. During this time, attending in-person appointments carried the additional risk of infection and may have been more difficult, particularly for parents when childcare centres and schools were closed and children required supervision. Patient behaviour during this time may not accurately reflect the long-term patterns observed in this analysis and future research focussing on post-pandemic behaviour would be valuable.

Finally, a significant limitation of this study is the high degree of heterogeneity observed among the primary studies. This variability can obscure the true effects and relationships being investigated, making it challenging to draw definitive conclusions. The diverse characteristics of the study population, such as differences in demographics, methodologies, and contextual factors, may introduce confounding variables that affect the reliability and generalisability of the findings. Alternative study designs such as a randomised control trial, or a series of trials in different clinical settings would contribute to a more definitive result in this review.

## Supplementary Information

Supplementary Material 1.

Supplementary Material 2.

## Data Availability

Data is provided within the manuscript or supplementary information files. Analysis data files are available on request.
