# Supplementary material for: Systematic review and meta-analysis of no show or non-attendance rates among telehealth and in-person models of care
Source: BMC Health Serv Res. 2025 May 9;25:663. doi: 10.1186/s12913-025-12826-2 (PMC12063363; doi:10.1186/s12913-025-12826-2)
Supplement: Supplementary file 2 — Supplementary Material 2. [file 12913_2025_12826_MOESM2_ESM.docx]

‘non* attend*’ OR (non AND attend*) OR ‘fail* attend*’ OR (failed AND attend*)’ OR ‘did not attend’

OR ‘failed to attend’ OR ‘no show*’ OR ‘non-appearance*’ OR ‘missed appointment*’ OR (missed

AND appointment*) OR ‘patient attend*’
